# Analysis of coumarin and angelica lactones in smokeless tobacco products

**DOI:** 10.1186/s13065-018-0506-2

**Published:** 2018-12-20

**Authors:** Kevin McAdam, Trevor Enos, Carol Goss, Harriet Kimpton, Arif Faizi, Steve Edwards, Christopher Wright, Andrew Porter, Brad Rodu

**Affiliations:** 1 Group Research & Development, British American Tobacco, Regents Park Road, Southampton, SO15 8TL UK; 2 3810 St. Antoine W, Montreal, QC H4C 1B4 Canada; 3 Department of Medicine, School of Medicine, University of Louisville, Room 208, 505 South Hancock Street, Louisville, KY 40202 USA

**Keywords:** Coumarin, Angelica lactone, Smokeless tobacco, Snuff, Snus

## Abstract

Differences in health risks between different styles of smokeless tobacco products (STPs) have prompted interest in their relative levels of toxic chemicals. We report here the development of methods for the analysis of STPs for coumarin and for α-angelica lactone (α-AL), both of which have been included in various published lists of tobacco toxicants. We have also determined the concentrations of these lactones in commercial STPs from the US and Sweden, representing 80–90% of the 2010 market share for all the major STP categories in these two countries: 65 products (plus two reference products) for coumarin and 66 commercial products for α-AL. For coumarin, methanol extracts of the STPs were analysed by HPLC/MS/MS. The lower limit of quantification (LOQ) and limit of detection (LOD) were, respectively, 100 and 30 ng coumarin/g of STP on a wet weight basis (WWB). Alpha-AL was determined via direct headspace GC/MS. The LOQ and LOD were 65 and 30 ng/g WWB respectively. Coumarin was detected In 3/33 Swedish snus, 5/13 US chewing tobaccos, 16/16 moist snuffs and 5/6 dry snuffs. Concentrations in those samples with quantifiable coumarin contents ranged from 186 to 1656 ng/g WWB. Concentrations of coumarin measured in this study were consistent with levels naturally found in tobacco. None of the STPs analysed would significantly contribute to coumarin exposure in consumers compared with dietary sources, and estimated exposure levels were 1000× lower than the European Food Safety Authority Tolerable Daily Intake. Hence the relevance of coumarin to the toxicity of STPs and its inclusion in the FDA’s list of harmful and potentially harmful compounds list is questionable. Measurements of α-AL in these STPs found that the majority did not have quantifiable contents, however, for three STPs concentrations of α-AL were above the LOQ (116–140 ng/g WWB) and for four other STPs concentrations of α-AL could be estimated between the LOD and LOQ. Beta-angelica lactone was tentatively identified in three of the STPs but the levels could not be reliably quantified. The levels of α-AL in tobacco products are reported here for the first time, but the relevance of α-AL to the toxicity of STPs is also highly questionable given that it has GRAS status as a permitted food additive.

## Introduction/background

Smokeless tobacco products (STPs) are widely used in the United States, Sweden, Norway and Asia. Although the International Agency for Research on Cancer (IARC), has collectively designated STPs as Group 1 carcinogens, i.e. carcinogenic to humans [1], evidence has been accumulating that health risks differ between STP categories. Some product styles such as Swedish snus and American CT have been shown to have lower health risks associated with their use [2] than other styles. As a result, there is substantial interest in comparing the chemical contents of different types of STPs [3]. In a 1992 review of the chemical composition of smokeless tobacco products Brunnemann and Hoffmann [4] compiled a list of 28 “carcinogenic agents in tobacco” which included coumarin, α-AL and β-AL. The same list was used by Hoffmann and Djordjevic [5] in a 1997 review of composition and carcinogenicity of smokeless tobacco and by the IARC in 2007 [1] in a table of “chemical agents identified in smokeless tobacco products”. There is increasing interest in regulation of tobacco products [6, 7]. The US Food and Drug Administration (FDA) has assembled a list of 93 harmful and potentially harmful constituents (HPHC) of tobacco products which may have to be reported [8]. This list covers both tobacco and tobacco smoke components and includes 79 that are designated as carcinogenic, as well as constituents that are respiratory toxicants, cardiovascular toxicants, reproductive toxicants or addictive. Coumarin is included in the FDA’s HPHC list because it is “banned in foods” in the US. In contrast to coumarin, α- and β-AL are not included in the HPHC list. In previously published research we investigated the potential presence in STPs of substances in the HPHC list including polycyclic aromatic hydrocarbons (PAH) [9], hydrazine [10], acrylamide [11], radioactive elements [12] and ethyl carbamate [13]. In the present study we determined the concentrations of two lactones, coumarin and α-AL, in several different styles of STP. We also report evidence for the presence of β-AL in a few of these STPs. Surprisingly, considering their inclusion in several lists of tobacco toxicant lists, these three lactones have not been identified as human carcinogens [14]. In fact at the time of writing this manuscript, α-AL is a permitted food ingredient in the European Union.

### Coumarin

**Fig. 1:**
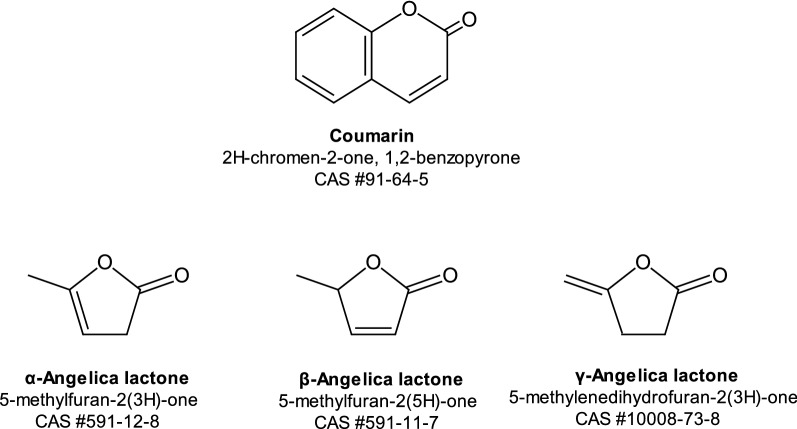
Structures of coumarin and α-, β- and γ-angelica lactone

Coumarin (2*H*-chromen-2-one, 1,2-benzopyrone) is a semi-volatile, low molecular weight lactone with a melting point of 71 °C and a boiling point of 302 °C [15]. The molecular structure of coumarin is shown in Fig. 1. Coumarin has a sweet odour similar to newly mown grass. It has been identified in over 60 plant varieties including vanilla leaf, parsnip, lavender, sweet clover and citrus oils [14]. Particularly high levels are found in tonka beans, cassia cinnamon, deertongue and sweet woodruff, as well as in essential oils such as cinnamon leaf (40,600 ppm) and bark oil (7000 ppm), cassia leaf oil and lavender oil [16]. Tobacco itself naturally contains coumarin [17–20] at relatively low levels (generally < 1 ppm), although fire-cured tobacco has been reported to contain higher levels of coumarin than other tobacco types [17].

Coumarin is used as a flavouring and fragrance material in common household and cosmetic products in the United States and Europe [14] and it is still used as a food and beverage ingredient in several countries. In the United States the use of coumarin as a food and beverage additive was restricted by the FDA in 1954. This was due to reports from manufacturers of pharmaceutical products that they would no longer supply coumarin or tonka bean for use in food due to hepatotoxicity observed in laboratory rodents fed high doses [21]. Food containing any added coumarin (itself or in the form of tonka beans or extracts) was deemed to be ‘adulterated under the act’ [22]. However there were no restrictions on the use of other flavouring agents such as vanilla extracts or cinnamon which also contain coumarin. Many of these plants and their extracts are currently permitted for use by the Food and Drug Administration (FDA) as food ingredients.

In 1985, based on carcinogenic and genotoxic concerns, the Codex Alimentarius [23, 24] specified maximum levels of 2 mg/kg of coumarin in foodstuffs and non-alcoholic beverages. In alcoholic beverages and certain caramel confectionary products, the permitted limit was 10 mg/kg and in chewing gum it was 50 mg/kg. The coumarin levels specified in the Codex Alimentarius were adopted into European law in 1988 [16, 25]. A revision in 2008 allowed 50 mg/kg for traditional and baked goods which referred to cinnamon in the labelling. Breakfast cereals were allowed 20 mg/kg and desserts 5 mg/kg. The European Food Safety Authority (EFSA) has determined a safe intake level of 0.1 mg/kg body weight (bw) per day [26, 27]. There are no regulations governing levels of coumarin in tobacco products, except in Germany [28]. In tobacco, particularly pipe tobacco, coumarin in the form of plant extracts and oils was used as a flavourant (fragrance) until about 1995. Since then, reports on the tumorigenicity of coumarin in rats eventually “resulted in coumarin being removed from the flavor formulations used throughout the tobacco industry on cigarette and pipe tobacco” [29]. However, some Asian and South American types of STP are flavoured with tonka beans, cinnamon and/or cloves and contain relatively high levels of coumarin (up to 17,400 ppm) [30, 31]. A clove cigarette tobacco [30] and tobacco from a cinnamon flavoured Indian bidi [32] also contained substantially higher levels of coumarin than are found in natural tobacco.

### Angelica lactones

The structures of the three isomers of angelica lactone viz. α-angelica lactone (α-AL) (5-methylfuran-2(3H)-one), β-angelica lactone (β-AL) (5-methylfuran-2(5H)-one) and γ-angelica lactone (γ-AL) (3-methylenedihydro-2(3*H*)-furanone) are shown in Fig. 1. They are naturally occurring compounds that are found in plants of the *Angelica* genus. α-AL is the predominant isomer followed by β-AL. The γ-AL isomer occurs at very low concentrations relative to the others [33]. Alpha-AL has also been found in raisins, white bread, soybeans and licorice and β-AL in wood smoke, roasted peanuts and almonds, raisins, burnt sugar and soybeans [33–35]. Both α- and β-AL have also been reported to occur naturally in tobacco [36–39], although their levels were never quantified. Alpha-AL is made commercially by dehydration of levulinic acid (from biomass carbohydrates) either by using an acid catalyst with distillation (to remove water) or by vacuum distillation [40, 41]. The reaction is reversible: addition of water converts α-AL back to levulinic acid. The isomers of AL can also be interconverted under specific conditions. For example when α-AL vapour at 200–300 °C and 25 mm Hg is passed over an activated clay, β-AL is formed in yields of up to 83% of the theoretical maximum amount [42].

Both α-AL (B.Pt. 167–170 °C) and β-AL (B.Pt. 208–209 °C at 751 mm Hg) are volatile compounds [43]. Alpha-AL, which has an odour variously described as like coconut, vanilla or chocolate [44] is traditionally used in perfumes. It is recognised by the Council of Europe, the US Flavour and Extracts Manufacturers Association and the US Food and Drug Administration. In the past, angelica root extract that contain the angelica lactones has been used as a tobacco additive, imparting a smoothing, caramel smoke taste [45, 46].

α-AL and β-AL have not been classified in terms of carcinogenicity to humans, and only a single study, which examined only one dose of β-AL (2 mg, twice per week), found a “weak carcinogenic” effect of β-AL in rats [47]. An earlier study by the same authors [48] using the same rat protocol, found α-AL to be non-carcinogenic. α-AL in fact has been shown to have anti-carcinogenic properties as it inhibits the formation of benzo[a]pyrene metabolite:DNA adducts in mice treated with benzo[a]pyrene [49], as well as inhibiting the formation of tumours in mice treated with benzo[a]pyrene [50].

The health risks from use of α-AL as a food flavourant were evaluated in 1999, at the 49th meeting of the Joint FAO/WHO Expert Committee on Food Additives (JECFA), using the Procedure for the Evaluation of Flavouring Agents. Based on the estimated per capita intakes of α-AL in the US and Europe and considering the fact that α-AL would be expected to be efficiently metabolised via commonly known biochemical pathways to innocuous products, the Committee concluded that the use of α-AL as a flavouring substance would not present a safety concern [51].

We are currently conducting a comprehensive survey of toxicants in an extensive and varied set of contemporary STPs from the United States and Sweden. Given the inclusion of coumarin and the angelica lactones in historic and/or current toxicant lists, the aims of the present study were to develop and validate methods for their analysis in tobacco products, and to quantify their levels in major STPs from the USA and Sweden.

## Methods and materials

### STP samples

For coumarin, a total 67 different STPs (65 commercial brands and two CORESTA reference products: CRP2, a moist snuff and CRP3, a dry snuff) were analysed. These are shown in Table 1. The commercial brands consisted of 8 loose snus (L snus) and 20 portion snus (P snus) from Sweden and 13 Chewing Tobacco (CT), 5 Dry Snuff (DS), 2 Hard Pellet (HP), 1 Soft Pellet (SP), 1 plug, and 15 moist snuff (MS) from the US (Table 1). For α-AL and β-AL, due to sample availability at the time of the study 66 commercial STPs were analysed: 9 L snus and 21 P snus from Sweden and 12 CT (plus one repeat measure of a CT sample), 5 DS, 2HP, 1 SP, 15 MS, and 1 plug), leading to 67 samples analysed. These are shown in Table 2. All the STP samples examined in this study were sourced in 2010. The Swedish products were obtained from Swedish retail websites, imported into the United Kingdom, and kept frozen at − 20 °C until analysis. The products represented seven different manufacturers and accounted for ~ 89% of the market share of STPs in Sweden in 2008 [9]. The US products were sourced from stores in the US, imported, and kept frozen at − 20 °C until analysis. The products represented nine different manufacturers and accounted for ~ 88% of the market share of STPs in the United States in October 2008 [9].

**Table 1:** Coumarin concentrations, WWB, and oven moisture, %, in STP brands

Brand	Style	Oven moisture (%)	Coumarin (ng/g) WWB (DWB)
Ettan	L snus	57.7	< LOD
General	L snus	57.0	< LOD
Goteborgs Rape	L snus	57.6	284 (670)
Granit	L snus	54.3	< LOD
Knox	L snus	56.6	< LOD
Kronan	L snus	57.3	< LOD
LD Original	L snus	55.8	< LOD
Skruf Strong	L snus	57.2	< LOD
Catch Licorice, mini	P snus	52.2	< LOD
Catch White Licorice	P snus	55.9	< LOD
CatchDry White Eucalyptus, mini	P snus	27.5	< LOD
Ettan	P snus	52.3	< LOD
General	P snus	54.8	< LOD
General mini	P snus	52.2	< LOD
General White	P snus	55.0	< LOQ
Goteborgs Rape	P snus	55.3	486 (1088)
Granit	P snus	53.7	< LOD
Granit White	P snus	44.7	< LOD
Grovsnus White	P snus	55.7	< LOD
Gustavus Original	P snus	N/D	< LOD
Knox	P snus	49.0	< LOD
Kronan	P snus	51.1	< LOD
LD Original	P snus	51.6	< LOD
Wise/oomph citrus menthol	P snus	9.6	< LOD
Romeo y Julieta Habanos	P snus	52.5	< LOD
Skruf Strong	P snus	52.3	< LOD
Tre-Ankare White	P snus	56.0	< LOD
1847 Original	P snus	47.5	< LOD
Beech Nut	CT	27.6	< LOD
Chattanooga	CT	24.3	< LOD
Durango	CT	25.9	< LOQ
Lancaster	CT	25.6	< LOD
Levi Garrett	CT	23.4	< LOQ
Morgans	CT	24.0	< LOD
Red Man Gold	CT	27.0	186 (254)
Red Man Regular	CT	27.0	< LOD
Southern Pride	CT	26.7	194 (265)
Starr	CT	26.1	< LOD
Stoker 707 Wintergreen	CT	23.8	< LOD
Taylors Pride	CT	24.0	< LOQ
Trophy	CT	24.9	< LOD
Bruton	DS	9.2	810 (892)
Dental Sweet	DS	9.5	< LOD
Garrett	DS	9	1656 (1820)
Honest	DS	8.7	1048 (1148)
Square	DS	8.6	1194 (1307)
CRP3	DS	8.4	330 (360)
Ariva Java	HP	3.8	< LOD
Stonewall Wintergreen	HP	4.9	< LOD
Oliver Twist Original	SP	18.9	< LOD
Copenhagen LC	MS	54.7	567 (1250)
Copenhagen Straight LC	MS	54.6	297 (654)
Grizzly Natural LC	MS	55.3	466 (1042)
Husky Natural FC	MS	56.1	296 (674)
Husky Straight LC	MS	56.9	206 (479)
Husky Wintergreen	MS	55.8	280 (634)
Kayak Straight LC	MS	53.3	386 (826)
Kodiak Straight LC	MS	54.3	973 (2130)
Kodiak Wintergreen	MS	52.8	454 (961)
Red Seal Natural FC	MS	55.2	376 (840)
Red Seal Natural LC	MS	56.5	584 (1342)
Silver Creek	MS	53.2	1033 (2207)
Skoal Straight	MS	55.4	500 (1120)
Timber Wolf Natural FC	MS	51.2	408 (836)
Timber Wolf Straight LC	MS	55.6	519 (1169)
CRP2	MS	54.5	265 (580)
Cannonball	Plug	21.2	< LOD

< LOD below limit of detection

< LOQ below limit of quantification

*WWB* Wet weight basis (as sold), *DWB* dry weight basis (calculated)

**Table 2:** α- and β-angelica lactone contents, WWB, of contemporary US and Swedish STPs

STP	Style	α-AL (ng/g WWB) Mean (RSD %, replicates)	Possible identification of β-AL
Swedish snus			
Ettan	L snus	< LOQ	
General	L snus	< LOQ	
Goteborgs Rape	L snus	< LOQ	
Granit	L snus	< LOQ	Yes
Grovsnus	L snus	36 (63.3)	
Knox	L snus	44 (29.3)	
Kronan	L snus	< LOQ	
LD Original	L snus	< LOQ	
Skruf Strong	L snus	< LOQ	
Catch Licorice, mini	P snus	< LOQ	
Catch White Licorice	P snus	< LOQ	
CatchDry White Eucalyptus, mini	P snus	< LOD	
Ettan	P snus	< LOQ	
General	P snus	57 (77.4)	
General mini	P snus	< LOQ	
General White	P snus	< LOQ	
Goteborgs Rape	P snus	< LOQ	
Granit	P snus	< LOQ	
Granit White	P snus	< LOQ	
Grovsnus	P snus	< LOQ	
Grovsnus White	P snus	< LOQ	
Gustavus Original	P snus	< LOQ	
Knox	P snus	< LOQ	
Kronan	P snus	< LOQ	
LD Original	P snus	< LOQ	
Oomph Citrus Menthol	P snus	< LOQ	
Romeo y Julieta Habanos	P snus	< LOQ	
Skruf Strong	P snus	< LOQ	
Tre-Ankare White	P snus	< LOQ	
1847 Original	P snus	< LOQ	
US STPs			
Beech Nut	CT	139 (44.5)	Yes
Chattanooga	CT	< LOQ	
Durango	CT	< LOQ	
Lancaster	CT	< LOQ	
Levi Garrett	CT	< LOQ	
Morgans	CT	< LOQ	
Red Man Gold	CT	< LOQ	
Red Man Regular	CT	< LOQ	
Southern Pride	CT	< LOQ	
Starr	CT	< LOQ	
Taylors Pride 1st sample	CT	39 (34.3)	
Taylors Pride 2nd sample	CT	< LOQ	
Trophy	CT	< LOQ	
Bruton	DS	< LOQ	
Dental Sweet	DS	< LOQ	
Garrett	DS	< LOQ	
Honest	DS	< LOQ	
Square	DS	< LOQ	
Ariva Java	HP	< LOQ	
Stonewall Wintergreen	HP	< LOD	
Oliver Twist Original	SP	140 (18.3)	Yes
Copenhagen LC	MS	< LOQ	
Copenhagen Straight LC	MS	< LOQ	
Grizzly Natural LC	MS	< LOQ	
Husky Natural FC	MS	< LOQ	
Husky Straight LC	MS	< LOQ	
Husky Wintergreen	MS	< LOQ	
Kayak Straight LC	MS	< LOQ	
Kodiak Straight LC	MS	< LOQ	
Kodiak Wintergreen	MS	< LOQ	
Red Seal Natural FC	MS	< LOQ	
Red Seal Natural LC	MS	< LOQ	
Silver Creek	MS	< LOQ	
Skoal Straight	MS	< LOQ	
Timber Wolf Natural FC	MS	< LOQ	
Timber Wolf Straight LC	MS	< LOQ	
Cannonball	Plug	116 (37.8)	

<LOQ indicates the sample did not contain quantifiable levels of α-AL during the a screening exercise

### Moisture analysis

Moistures of the STPs were determined using a gravimetric oven moisture method [52].

### Coumarin analysis

The method was based on a previously published report of the analysis of coumarin in mainstream tobacco smoke using HPLC/MS/MS [53].

#### Sample preparation

Other than removing the P snus tobacco from its sachet/pouch, no sample milling or processing was carried out prior to analysis.

#### Reagents

Coumarin standard was obtained from Sigma-Aldrich (Gillingham, UK) and Fisher Scientific (Loughborough, UK). HPLC-grade methanol and AR grade formic acid were obtained from Fisher Scientific. Water was deionized by an Elga Pure Lab Ultra system (resistivity not < 18.2 MΩ cm).

#### Stock solutions

A coumarin stock standard (~ 100 mg/l) was prepared by dissolving 50 mg coumarin in 500 ml of 50% methanol/water (v/v). A series of calibration standards (~ 5 ng/ml to ~ 1000 ng/ml) were prepared by dilution of the stock standard. All standard solutions were stored in screw-cap vials at 4–5 °C.

#### Solvent extraction

Approximately 1.0 g (0.99–1.01 g) of the STP was accurately weighed out into a 50 ml centrifuge tube (Fisher Scientific). 4 ml of water were added and the mixture was equilibrated for 16 h at room temperature. 10 ml of methanol were added and the mixture was macerated (Polytron PT3100, Kinematica AG) at 10,000 rpm for 1 min. The suspension was sonicated at 40 °C for 10 min and shaken (KS501 Flatbed Shaker, Janke and Kunkel) for 30 min at 100 rpm. After centrifuging at 4600 rpm for 5 min, the supernatant was transferred to a 40 ml amber vial and the remaining solvent was squeezed out using a syringe (Discardit 20 ml, BD) and PTFE filter (GD/XP 25 mm, 0.45 μm; Whatman). A second extraction using 5 ml of methanol was carried out in the same way. The first and second extracts were combined and transferred to a tube labelled “extract” and 5 µl were injected into the HPLC/MS/MS.

#### HPLC analysis

The compounds in the sample were separated by HPLC using a 1200 series LC system (Agilent Technologies) consisting of a degasser, a binary pump, an autosampler and a column heater operated at 40 °C. The system was equipped with a Luna C18 analytical LC column (100 mm × 2 mm; i.d., 3 µm; Phenomenex, UK). The detector was an Applied Biosystems API 5000 triple quadrupole mass spectrometer.

Mobile phase A was prepared by dissolving 1 ml formic acid in 1 l water to give a 0.1% formic acid solution in water. Mobile phase B was prepared by dissolving 1 ml formic acid in 1 l methanol to give a 0.1% formic acid solution in methanol. The sample injection volume was 5 µl and the HPLC flow rate was set at 0.3 ml/min with the following elution profile (gradient): 20% B at 0 min; increasing linearly to 60% B at 4 min, and then to 100% B at 4.1 min; remaining at 100% B until 5.0 min, dropping to 20% B at 5.1 min, and remaining at 20% until 8 min. The typical HPLC column backpressure was 230 bar.

#### MS/MS parameters

Positive mode electrospray ionisation (ESI) was used in the analysis. The transition used for the quantitation of coumarin was the [M+H] + ion of 147 → 103 amu. The curtain gas, gas 1(GS1) and gas 2(GS2) flows were all set to 50 psi, and the source temperature was 650 °C. The declustering potential was 166 V and the collision energy applied was 25 eV.

#### Validation

The method was validated using seven types of STP: LD Original (P snus and L snus), Redman Gold (CT), Square (DS), Oliver Twist (SP), Skoal Straight LC (MS) and Cannonball (plug). The recovery, repeatability, accuracy and bias were all within 85–115% of the target concentration. The lower limit of quantitation was established as 100 ng/g STP (or 5 ng/ml extract), based on a signal to noise ratio (S/N) of > 10, and the upper limit of quantitation was 19 µg/g STP (or 1000 ng/ml extract). The lower limit of detection was established as 30 ng/g STP, based on an S/N of > 3.

### Angelica lactone analysis

Analyses were conducted by the Food and Environment Research Agency (York, UK).

***Challenges in analysis of angelica lactones:***

Initially a SPME method was developed. During early method development using SPME, an α-angelica lactone standard was prepared in methanol and analysed by direct splitless injection. Three main peaks were observed at *m/z* 98 and 55. The first peak corresponded to α-AL. The second peak with an almost identical fragmentation pattern to α-AL was thought to be β-AL. However this could not be unequivocally identified due to difficulties in obtaining a β-AL reference standard. (Identification of β-AL in the STP samples is discussed later). A third peak was observed with an identical fragmentation pattern to the methyl ester of levulinic acid indicating that α-AL reacts with methanol to form methyl levulinate. Addition of water to the STP extract and heating (for 5 min at 70 °C) in a headspace vial completely eliminated the a α-AL peak, but the expected product, levulinic acid, was not observed. Hence, to avoid analytical artefacts arising from reactions of the analytes with these reagents, extractions were carried out in dichloromethane with a magnesium sulphate desiccant to remove residual moisture. However, possibly due to matrix interference, the SPME method still gave inconsistent results and a direct headspace GC/MS method was used instead for analysis of α-AL in this work, as described below.

### Analysis of α-AL

#### Sample preparation

Sample preparation was kept to a minimum in order to minimise losses of the volatile α-angelica lactone from STPs pre-quantification. The pouches of P snus were opened and the tobacco was emptied into 40-ml Nalgene screw-cap bottles. No preparation was required for the L snus and MS products, which comprised fine shreds of tobacco, and for the DS which was a fine dry powder. The CT products, which consisted of compressed leaves, were cut into 1–3 mm pieces with scissors. The SP product comprised compressed pelleted leaves in vacuum packs. Three of these were opened, unrolled and chopped in a domestic food blender into 1–3 mm pieces. For the plug product, a portion of the compressed block was removed and chopped in a domestic food blender into 1–3 mm pieces.

#### Reagents

Acetophenone-d3 (AP-d3; 99 atom% D), benzophenone-d10 (99 atom% D), sodium sulphate (anhydrous powder, > 99%), magnesium sulphate (anhydrous powder, > 99%) and a standard of α-AL (98%) were obtained from Sigma-Aldrich (Gillingham, UK). Dichloromethane (DCM), HPLC grade, was obtained from Fisher Scientific (Loughborough, UK). Supelco screw-cap 10-ml glass round-bottomed HS vials and caps were obtained from Sigma-Aldrich.

#### Stock solutions

Stock standards of α-AL and AP-d3 (~ 2000 mg/l) were prepared by dissolving ~ 100 mg in 50 ml of DCM. Separate working standard solutions (~ 40 μg/ml) were prepared by dilution of stock standards with DCM. A series of α-AL calibration standards were prepared in DCM by appropriate dilution of the working standard at ~ 0 (DCM only), 1.0, 2.0 and 4.0 mg/l. An internal standard (IS) solution of AP-d3 (~ 2 mg/l) was prepared by diluting the AP-d3 working standard with DCM. All standard solutions were stored in screw-cap vials at 4–5 °C.

#### Direct headspace

Each STP sample (250 ± 10 mg) was weighed directly into a 10-ml headspace vial and 100 ± 5 mg magnesium sulphate was added. The contents were mixed thoroughly using a fine-bladed spatula, sealed securely with a vial screw-cap and either allowed to stand for 1 h with occasional swirling or placed on a roller-mixer. Working rapidly, the vial cap was then removed, 25 μl of AP-d3 IS (2 mg/l) was added, and the vial resealed tightly. Where required, standard addition of α-AL solution was carried out in the same way. The contents were mixed for 1 h prior to analysis by HS-GC–MS by gentle swirling such that they did not touch the inner surface of the cap or septum. The samples were either analysed immediately or stored at − 18 °C for a maximum of 24 h until required. After attaining room temperature, sample vials were incubated for 5 min at 70 °C, and 1.0 ml of headspace gas withdrawn, injected into the GC–MS and monitored in SIM channels m/z 98 (α-AL) and m/z 123 (AP-d3).

#### GC–MS analysis

The system comprised a CTC Analytics Combi-PAL autosampler (CTC Analytics, Zwingen, Switzerland) fitted with a 2.5-ml syringe maintained at 75 °C. A ThermoQuest Trace 2000 GC (Thermo Scientific, Loughborough, UK) was used with splitless injection at 250 °C with a splitless time of 0.75 min and a split flow of 50 ml/min. The injection volume was 1.0 ml. A 30-m × 0.25-mm i.d. × 0.25-μm film thickness Zebron ZB-WAX column (Phenomenex, Macclesfield, UK) column was used with helium carrier gas at a constant flow rate of 1 ml/min. The GC oven was programmed with an initial temperature of 35 °C, increased to 135 °C at 5 °C/min, and then to 240 °C at 40 °C/min.

MS detection was achieved using a Voyager GC/MS (Thermo Scientific, Loughborough, UK) with electron ionization at 70 eV in selected ion monitoring mode. Data were acquired at m/z 43, 55, 70, 77, 98 and 123 for α-AL, and at m/z 77 and 123 for AP-d3. Full-scan mode was used on occasion to identify specific GC peaks.

**Fig. 2:**
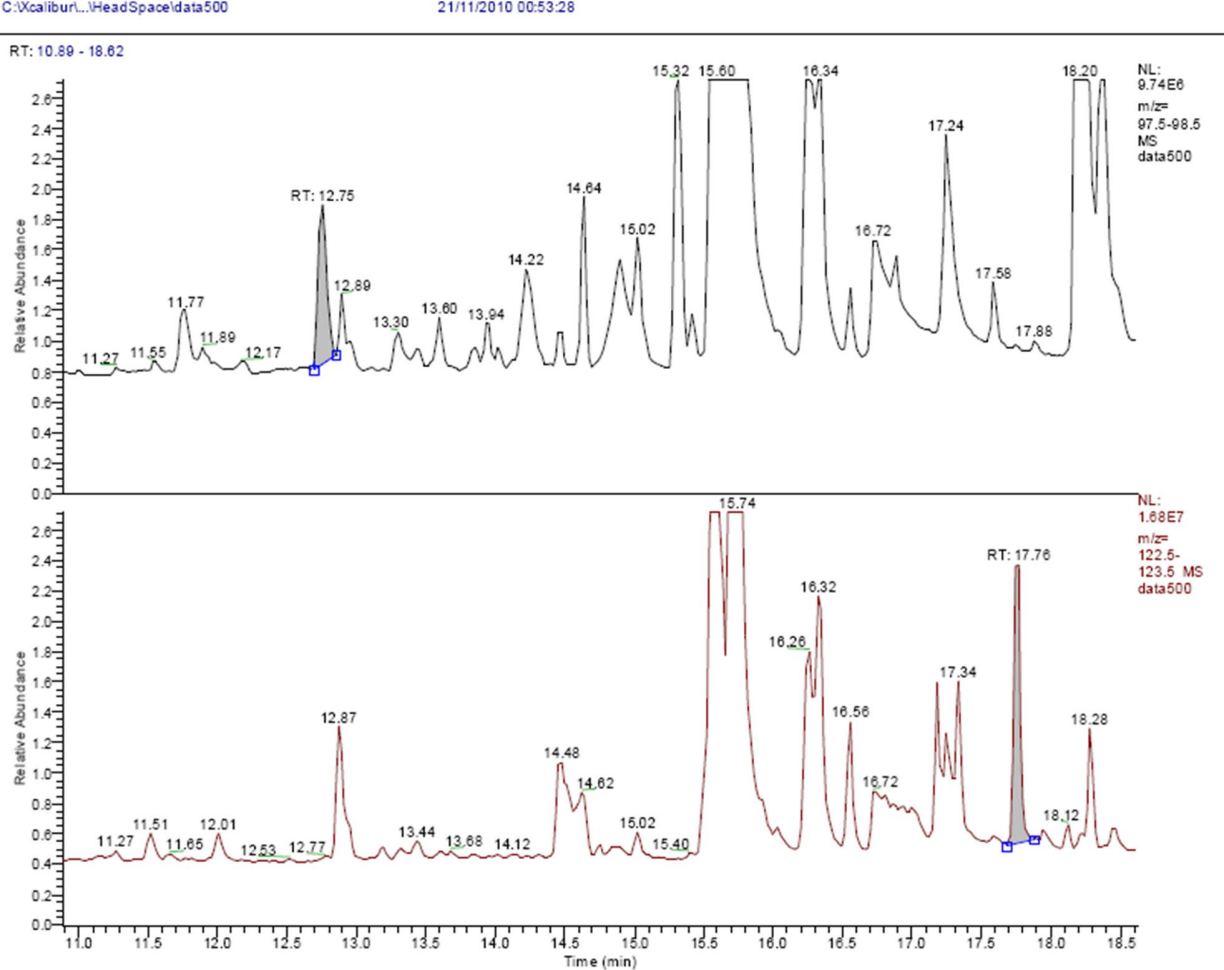
HS-GC–MS chromatogram of an STP spiked with 86 ng/g of α-AL

The retention time for α-AL was 12.75 min, and m/z 98 was used for quantitation. A sample chromatogram is shown in Fig. 2.

#### Validation

The linear dynamic range of the method was evaluated by standard additions and the limit of detection (LOD) and limit of quantification (LOQ) were evaluated using seven STPs to represent the different product types in the study: Gustavus Original (P snus), LD loose (L snus), Redman Regular (CT), Square (DS), Kayak Straight LC (MS), Oliver Twist (SP) and Cannonball (plug).

##### Standard addition

Four sample replicates were prepared as above, with the addition of 25 μl of the 0, 1.0, 2.0 or 4.0 mg/l α-AL calibration standard and 25 μl of IS solution (2 mg/l). The areas of the peaks attributable to α-AL and IS in each of the four vials were measured by integration and the ratio of the peak areas (α-AL/IS) was plotted against the equivalent concentration of α-AL in the sample expressed as μg/kg. The concentration of α-AL in the sample was calculated from the *x* axis intercept extrapolated from the standard addition calibration line.

##### LOD and LOQ

The LOD and LOQ were calculated via an established procedure [54] based on the mean (μB) and standard deviation (σB) of the response level for the zero standard addition, where: LOD = μB + 3σB = 30 μg/kg, and LOQ = μB + 10σB = 65 μg/kg.

#### Determination of α-AL in the STP samples

All samples were first analysed, in triplicate, in an initial semi-quantitative screening exercise. During this exercise, chromatograms containing a peak at the same retention time as the α-AL standard peak, with a peak area greater than that of the reagent blank, and with a peak area ratio to the IS of ca. 0.1 or greater, were considered positive and were taken forward for quantitative analysis by standard addition. Where there were inconsistencies between triplicate analyses, sampling was repeated. If samples were found to contain a relatively large a-AL peak during screening (i.e. exceeding the calibration range), a smaller sample size of 50 mg was taken for standard addition analysis while maintaining the same volume and concentration of standard and IS, thereby increasing the relative ratio of the added standard. Thus, for 250 mg sample weights the standard addition range was approximately 0, 100, 200 and 400 mg/kg depending upon the exact concentration of the prepared working standard. This was later modified to approximately 0, 50, 100 and 200 mg/kg for improved accuracy. For 50 mg samples, the standard addition range was extended to approximately 0, 200, 500 and 1000 mg/kg.

For some of the STPs the chromatograms contained peaks from other volatiles that co-eluted with, or eluted very closely to, the α-AL or IS peaks, to the extent that the peak area ratios could not be calculated. In these cases, the absolute peak area was first examined and compared directly with the peak areas obtained for the reagent blank and spike, and, if significantly higher, compared with the absolute peak areas of samples analysed in the same batch which were screened positive and subsequently analysed quantitatively. Also, as expected, sample matrix effects were inconsistent across the sample range and the MS response was very variable between samples as evidenced by the variability in results from those samples analysed quantitatively.

### Analysis of β-AL

As no β-AL standard could be sourced, validation could not be conducted on this analyte and it was not possible to include it within the scope of the method directly. Several minor peaks (impurities) were present in the α-AL reference standard. To ascertain if one of these was due to β-AL, a concentrated α-AL standard was analysed using full scan MS. A peak eluting at ca 17 min had a mass spectrum consistent with the NIST spectrum of β-AL [55], characterized by the presence of a fragment ion at m/z 83, which is more pronounced in the mass spectrum of β-AL than that of α-AL. Hence for analysis of the STPs an additional SIM channel m/z 83 was used to monitor for the presence of β-AL in addition to the ions common to both isomers i.e. m/z 98, 70 and 55. While all of the samples were screened for β-AL, not all of them were carried out in triplicate, because the NIST mass spectrum for β-AL was not identified until approximately half of the screening analyses had been completed. Since no standard addition could be carried out for this analyte, chromatograms containing a peak corresponding to the retention time of β-AL were screened in a similar way to α-AL and those judged to have peak areas large enough to warrant further investigation were taken forward for further analysis.

## Results and discussion

### Coumarin

**Fig. 3:**
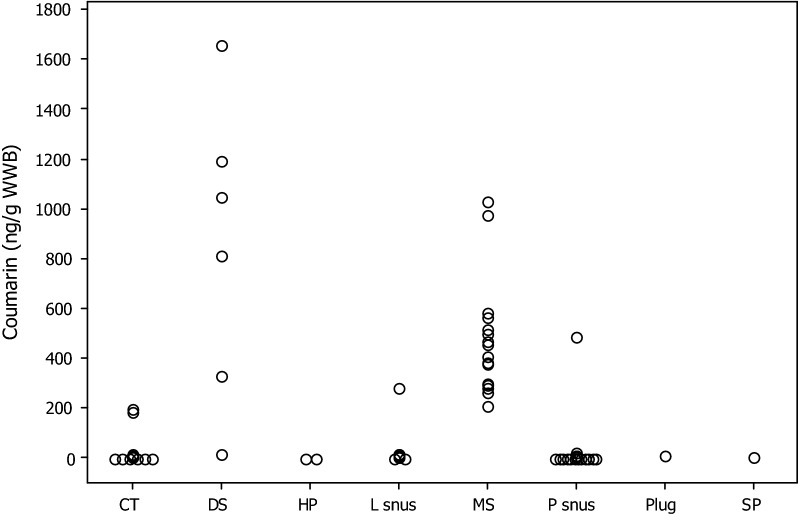
Individual value plot: coumarin concentrations (ng/g STP WWB) by STP style. Concentrations < LOD or < LOQ are shown as 0

**Fig. 4:**
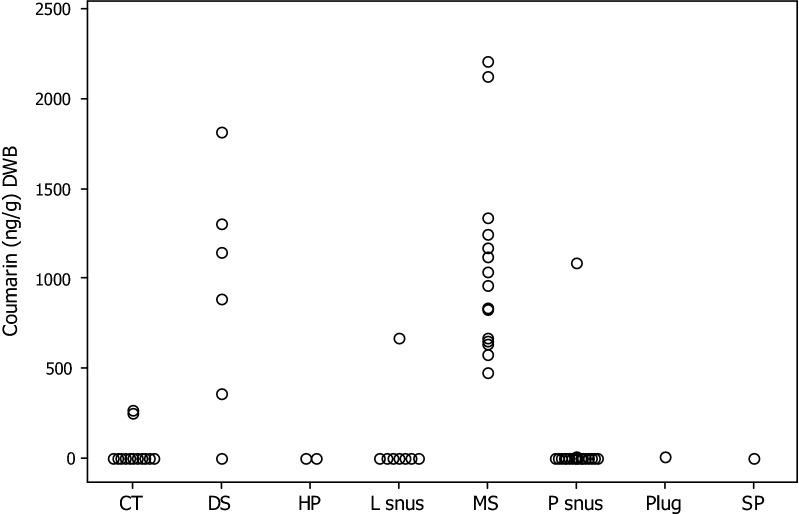
Individual value plot: coumarin concentrations (ng/g STP DWB) by STP style. Concentrations < LOD or LOQ are shown as 0

The WWB concentrations of coumarin for the 67 STPs sample are shown in Table 1 together with their moisture contents and calculated DWB concentrations. Individual value plots for both WWB and DWB coumarin concentrations are shown in Figs. 3 and 4. Of the 65 brands and two reference products tested, 29 samples containmed detecteble levels, of which 25 contained quantifiable levels of coumarin. Levels of coumarin in the HP, SP and plug products were < LOD. Only 2 of the 28 Swedish snus products contained quantifiable levels of coumarin: Goteborgs Rape loose (284 ng/g WWB) and Goteborgs Rape portion (486 ng/g WWB). All of the other Swedish snus samples contained levels of coumarin < LOD, except for one that was < LOQ. For the CT brands 2 of the 13 had quantifiable levels of coumarin: Red Man Gold (186 ng/g WWB) and Southern Pride (194 ng/g WWB). For DS, five of the six samples contained coumarin levels in the range 330–1656 ng/g WWB, while one was < LOD. All of the 16 MS samples contained quantifiable levels of coumarin in the concentration range 206–1033 ng/g WWB. On a WWB, levels of coumarin were significantly higher in DS than in MS. But when expressed on a DWB, there was no significant difference (at P < 0.05) between MS and DS coumarin levels, suggesting that the coumarin in these STPs originated from the tobacco.

### Sources of coumarin in tobacco

Given the differing levels of coumarin between STP styles, and in some cases within styles, the question arises as to whether the levels are intrinsic to the tobaccos used in the product or whether flavour materials containing coumarin have been added.

The natural coumarin content of tobacco can be inferred from published work, which is summarised in Table 3. Various levels have been reported in “natural” tobaccos. Fujimori et al. [18] reported 600 ng/g of coumarin in a sample of air-cured Burley tobacco containing 9% moisture. Tobaccos from 68 brands of US cigarette were tested for coumarin in 1999 [20]. Of these 67 contained coumarin levels < LOD (i.e. < 13 ng/g WWB) while one contained 390 ng/g WWB of coumarin. Levels of coumarin in two US MS and two US cigar tobaccos were reported as < LOD (i.e. < 380 ng/g) on a WWB, although no moisture values were given [30]. Christakopoulis et al. [17] determined coumarin levels in 28 different “natural” tobaccos from several countries. The tobaccos included flue-cured, air-cured, sun-cured and fire-cured types. Most of the tobaccos had coumarin levels of < 1000 ng/g DWB, while the fire-cured tobaccos generally had higher levels (1000–4700 ng/g DWB).

**Table 3:** Levels of coumarin in tobacco and tobacco products

Tobacco type or product	Country	Samples with coumarin/total samples	Level (ng/g)	Comments	References
Flue-cured	Brazil, Malawi, Zimbabwe, US	11/12	< LOD-900	Results on DWB, LOD 50 ng/g	Christakopoulos et al. [17]
Air-cured	Poland, Italy, Zimbabwe, US	6/8	250–1300		
Sun-cured	Greece, Turkey	1/2	< LOD-250		
Fire-cured	Poland, US	6/6	1600–4200		
Burley	US	1/1	600	Result on WWB (9% water)	Fujimori et al. [18]
Zarda	SE Asia	2	3.8 × 10^5^, 4.4 × 10^5^	Results on WWB LOD 380 ng/g	Lisko et al. [30]
Qiwam	SE Asia		1.9 × 10^5^		
Snuff	US	0/2	< LOD		
Clove cigarette filler	US	1/2	< LOD-4600		
Cigarette filler	US	1/68	< LOD-390	Results on WWB LOD 13 ng/g	Stanfill and Ashley [20]
Bidi filler	India (from US market)	1/17	< LOD-3.6 × 10^3^	Results on WWB LOD 200 ng/g	Stanfill et al. [32]
Rapé snuff	Brazil	9/11 tobacco containing	< LOD-2.8 × 10^6^	Results on WWB Several contained tonka bean	Stanfill et al. [31]
		2/2 non-tobacco	5.8 × 10^6^ − 1.7 × 10^7^		

In comparison, our study found that, when expressed on a DWB, coumarin levels exceeded 1000 ng/g for one of the Swedish snus products (1088 ng/g), 3 of the 6 DS products (1148, 1307 and 1820 ng/g) and 7 of the 16 MS products (1033 ng/g).

DS and MS typically contain high levels of fire-cured tobacco [28] together with some air-cured tobaccos, while CT generally contains only air-cured tobaccos. Spices and flavours including cinnamon [56] or tonka bean [28] have also been used in the formulations, but it is not known if STPs analysed in this study contain coumarin related spices or flavours.

#### Correlation with PAH

**Fig. 5:**
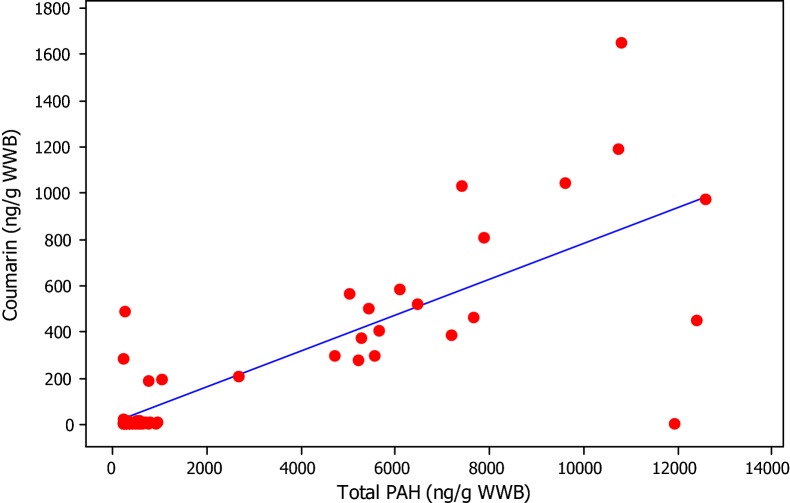
Coumarin vs total PAH in STPs (r = 0.808, P = 0.000)

Christakopoulis et al. [17] hypothesized that smoke from the hickory wood used in fire-curing tobacco contributes to the relatively high coumarin levels found in these tobacco styles. Coumarin has been identified in smoke from softwoods and hardwoods [57–59] and DS and MS contain large proportions of fire-cured tobacco [28]. Since fire-cured tobaccos also contain polycyclic aromatic hydrocarbons (PAH) derived from the wood-smoke used during curing, we would expect a correlation between PAH and coumarin levels in the STPs if fire-curing was the source of both contaminants. We therefore compared our coumarin data with levels of total PAH reported by McAdam et al. [9] for the same STP brands. (It should be noted that samples for PAH testing were obtained in 2008 while samples for coumarin testing were obtained in 2010, and therefore some differences in contents might arise due to product variability). A plot of coumarin vs total PAH concentrations is shown in Fig. 5. The Pearson correlation, r, is 0.808 with P < 0.001. This is consistent with PAH and coumarin being derived from the same source i.e. fire cured tobaccos in the STP tobacco blend.

### Exposure to coumarin from STP use

Exposure of the consumer to coumarin from use of STPs will depend on its concentration in the STP, the rate of consumption of the STP by the consumer and the proportion of coumarin extracted from the STP during use. In the case of snuffs and chewing tobaccos, the amount of expectoration that occurs with use must also be considered.

#### Daily consumption

For Swedish snus, Andersson et al. [60] found the average daily consumption was 14.4 g snus/day among 23 P snus users, and 20.8 g snus/day among 22 L snus users. In a much larger study [61] 2914 Swedish snus users reported average daily consumptions of 11–12 g/day for P snus and 29–32 g/day for L snus.

For US MS, which is similar in terms of moisture and usage to Swedish L snus, reported consumption rates vary widely. Maxwell [62] estimated average MS consumption amongst US users in 1980 as 7.3 g/day (one and one-half 1.2 oz cans per week). The Surgeon General’s 1986 report on smokeless tobacco “assumed” a consumption rate for MS of 10 g/day [63]. In 1988 Hatsukami et al. [64] reported average consumption of 12.4 g/day amongst 56 male adult consumers of a US MS product, but there was a high variability among the users. Hecht et al. reported an average consumption of 4.2 tins/week (or 20.4 g/day for 1.2 oz tins) of MS (mainly Copenhagen, Skoal and Kodiak brands) among 182 [65] and 212 STP users [66]. Among a group of 15 users, Hecht et al. [67] reported a considerably lower consumption of 1.1 ± 0.8 tins/week (or 5.3 g/day).

#### Extraction

There are no reports of the amount of coumarin extracted from an STP during use. However values for a range of constituents of Swedish snus have been published [68]. The most water soluble constituents such as nicotine, propylene glycol, TSNAs and inorganic ions, had mean extractions ranging from 24 to 38% after 1 h of use. Geraniol, which has a similar solubility to coumarin, had an extraction rate of about 24%, which is the figure we have used for coumarin.

#### Expectoration

The amount of expectoration during MS use was quantified as part of a study of NNK uptake in 15 STP users [67]. The subjects were given 2 g samples of MS contained in a pouch. The pouches were held in the mouth for 30 min and the expectorated saliva and used pouches were collected and analysed for NNK. The proportion of NNK in the expectorated saliva averaged 14.2% of the total NNK extracted from the MS. However the inter-subject variability was very large with percentages of NNK lost by expectoration ranging from 0 to 48.7%. This is, to our knowledge, the only study of toxicant losses due to expectoration.

#### Exposure

We have estimated maximum exposures to coumarin from use of STPs using the concentrations of coumarin found in the present study, together with the highest consumption estimates from the literature and an estimated extraction efficiency for coumarin of 24%. These are tabulated in Table 4.

**Table 4:** Estimated exposures (ng/person/day) to coumarin from Swedish and US STPs

STP	Coumarin concentration by brand (ng/g)		STP consumption (g/day)	Extraction in mouth (%)	Exposure (ng/day) (using highest reported consumption rate)	
	Min	Max			Min	Max
Swedish P snus	< 30	486	14.4^b^, 11–12^c^	24	< 104	2288
Swedish L snus	< 30	331	20.8^b^, 29–32^c^	24	< 230	2542
US MS^a^	206	1033	7.3^d^, 10^e^, 12.4^f^, 5.3^g^, 20.4^h^	24	613^a^	5057^a^
US DS	< 30	3347	N/A	24	N/A	N/A
US CT	< 30	247	N/A	24	N/A	N/A

*N/A* not available

^a^ Actual exposures may be lower due to expectoration

^b^ Andersson et al. [60]

^c^ Digard et al. [61]

^d^ Maxwell [62]

^e^ Surgeon General [63]

^f^ Hatsukami et al. [64]

^g^ Hecht et al. [67]

^h^ Hecht et al. [65]

The vast majority of Swedish snus users would have minimum exposure to coumarin < 104 and < 230 ng/day for P snus and L snus respectively. Users of the one P snus and one L snus brand with coumarin levels > LOQ would be exposed to 2288 and 2542 ng/day respectively. Users of US MS would be exposed to between 613 and 5057 ng/day using the consumption rate of 20.4 g/day [65] and with no expectoration. Lack of consumption figures for US DS and CT prevented calculating exposures for these STPs, but exposure for DS is likely to be of the same order of magnitude to that found with MS. For a 60 kg person the maximum exposures to coumarin from STPs by category will be: P snus 38.1 ng/kg bw/day; L snus, 42.4 ng/kg bw/day, and MS 51.2 ng/kg bw/day.

The maximum daily human exposure to coumarin from dietary sources and fragrance use in cosmetic products has been estimated as 6 × 10^4^ ng/kg/day for a 60-kg consumer [16]. The largest source of exposure to coumarin is believed to be the use of cassia cinnamon as a flavourant [16]. Even for consumers of STPs with the highest levels of coumarin found in this study estimated exposures are three or more orders of magnitude lower than exposures from dietary and fragrance sources. They are also lower still than the EFSA safe intake level of 1 × 10^5^ ng/kg of body weight per day [27]. We therefore conclude that it is unlikely that STP use creates any significant risk to the user from coumarin exposure.

### Angelica lactones

#### α-AL

The screening and quantitative results for α-AL are given in Table 2. The initial screening exercise identified 57 samples that did not contain quantifiable levels of α-AL, and these STPs are labelled “<LOQ” in Table 2. In the standard addition quantitiative experiments three samples were identified that contained α-AL above the LOQ (65 ng/g) ranging from 116 to 140 ng/g; these were the SP product (Oliver Twist Original—140 ng/g, RSD 18%), a CT product (Beech Nut—139 ng/g, RSD 44%) and the plug (Cannonball—116 ng/g, RSD 37%). The relatively high variability in the Cannonball plug and Beech Nut CT may in part be attributed to sample inhomogeneity. The plug and CTs consisted of large cut leaves or compressed tobaccos that were relatively difficult to prepare as homogeneous sub-samples without compromising analyte integrity. In contrast, most other STPs comprised relatively fine materials from which homogeneous samples were easier to prepare. For two of the STPs (Stonewall Wintergreen and CatchDry White Eucalyptus, mini portion) the chromatograms contained peaks from other volatiles that made it impossible to calculate the peak area ratios. For these STPs concentrations of α-AL were estimated as described in the “Method” section; however the resulting α-AL concentrations were considered to be below the LOD.

The results of the screening analysis revealed that four STPs gave α-AL/IS peak area ratios close to 0.1, and were subsequently analysed by standard addition. All of these samples contained α-AL levels below the LOQ (65 ng/g) but above the LOD (30 ng/g) and were thus deemed to contain trace amounts of the analyte (Table 1): α-AL concentrations of these samples are presented in Table 2, although the values are below the formal LOQ. Taylors Pride (39 ng/g, RSD 34%), Grovsnus L snus (36 ng/g, RSD 63%), General P snus (57 ng/g, RSD 77%) and Knox L snus (44 ng/g, RSD 29%). These are obviously imprecise estimates with large variabilities which reflect how close the concentrations are to the LOD of the method. As evidence for this, a second sample of Taylors Pride was run through the screening approach and did not show quantifiable levels of α-AL (<LOQ).

#### β-AL

Of the samples analysed, Granit L snus, Beech Nut and Oliver Twist showed peaks in their chromatograms that were consistent with the presence of β-AL (Table 2). The MS ion abundance profiles were considered to be clearly acceptable for only one sample (Oliver Twist). For Granit L snus and Beech Nut, the MS ion profiles were inconclusive. Given the lack of a β-AL standard it was not possible to quantify β-AL in the samples. If it is assumed that α-AL and β-AL have the same MS responses, then the concentrations of β-AL can be estimated from the area of the β-AL peak relative to the α-AL peak. With this assumption, the concentrations of β-AL for the three STPs were in the approximate range 100–200 ng/g. However, it must be emphasized that this process only provides a crude and potentially unreliable estimate of the β-AL content.

#### Origin of α-AL and β-AL in tobacco

α-AL and angelica root have in the past been included in lists of flavourants that have been, or could be, used in tobacco [45]. However, it is not known if α-AL or α-AL-containing flavours are currently added to STPs. Without knowledge of levels of α-AL in “natural” tobacco it is not possible to rule out the presence of α-AL-containing flavours in the STPs where it was detected.

α-AL is generated during the caramelisation of reducing sugars such as fructose and sucrose [69]. α-AL and β-AL have been reported to be formed by acid catalysed dehydration of fructose solutions at elevated temperatures [70]. Whether these reactions are sufficiently rapid at the temperatures used to cure tobacco is not known. α-AL is also a component of wood smoke [71] and hence could be present in fire-cured tobacco as has been proposed for coumarin.

However, the possibility that α-AL and β-AL are generated as artefacts during the extraction and analysis of tobacco cannot be ruled out. The dehydration of fructose to form α-AL and β-AL [70] has been shown to occur during steam distillation extraction (SDE). For example Caven-Quantrill and Buglass [72] generated extracts from grape juice either at elevated temperatures using SDE or at ambient temperatures using stir bar sorptive extraction (SBSE). The SDE extracts contained sugar degradation products such as acetylfuran, furfural, 5-methylfuran, furyl hydroxymethyl ketone and α-AL and β-AL. But these products were not observed in the SBSE extracts.

The artefactual formation of α-AL or β-AL during steam distillation may explain why Lloyd et al. [37] found that steam distillates of tobacco contained α-AL and β-AL while chloroform extracts of tobacco obtained at ambient temperature and purified by molecular distillation had no detectable levels. Weeks et al. [39] also used steam distillation to prepare the tobacco extracts in which they reported the presence of α-AL and β-AL.

We have already noted that α-AL is not stable in the presence of water and can undergo hydrolysis to levulinic acid, which is readily esterified by alcohols. Indeed, the methyl and ethyl esters of levulinic acid were observed in the present study when methanol and ethanol were investigated as extraction solvents, which is why dichloromethane was eventually chosen for extraction. Anhydrous magnesium sulphate was also added to the extraction mixture to remove residual water from the STP samples. However there is the additional possibility that α-AL, β-AL and γ-AL can interconvert during GC sample introduction by heated injection. Zviely et al. [33] reported that the relative proportions of the AL isomers, each of which had a different GC retention time, changed depending on the GC conditions used. They found that acidic column materials tended to increase the isomerisation of α-AL to β-AL.

#### Relevance of the angelica lactones to STP toxicity

The inclusion of α-AL and β-AL in published lists of STP carcinogens [1, 4, 5] is curious. As stated in the introduction there is no evidence that α-AL is carcinogenic in either animals or man. In fact it is a GRAS (Generally Recognised as Safe) flavour component and there is very limited evidence suggesting that β-AL is carcinogenic in animals, but no evidence that it is a human carcinogen.

Exposure to α-AL can be estimated in the same way as for coumarin. None of the users of the brands of Swedish snus, MS, DS or HP tested in this study would be exposed to more than 65 ng of α-AL per gram STP used. Only one of the 13 CTs tested and the plug and soft pellet products had more than 65 ng/g of α-AL. Only three of the STPs examined showed evidence that they contained β-AL, and for two of these the evidence for the presence of β-AL was deemed to be inconclusive.

It would appear that, considering their toxicological profiles and low concentrations, the angelica lactones would not present a significant risk to STP consumers.

## Conclusions

In this study a wide range of STPs from Sweden and the US were analysed for the lactones coumarin and angelica lactones. Of the 65 brands and two reference products tested, 25 had quantifiable levels of coumarin. Coumarin concentrations varied with the style of STP: levels of coumarin in the HP, SP and plug products were < LOD, and only 2 of the 28 Swedish snus products had quantifiable levels of coumarin (at 284 and 486 ng/g WWB). All of the other Swedish snus brands (except one < LOQ) had levels of coumarin < LOD. For the CT brands 2 of the 13 had quantifiable levels of coumarin (at 186 and 194 ng/g WWB). All of the 16 MS samples had quantifiable levels of coumarin ranging from 206 to 1033 ng/g WWB. For DS, five of the six samples had coumarin levels in the range 330–1656 ng/g WWB, while one brand was < LOD. WWB levels of coumarin were significantly higher in DS than in MS, but after correction to DWB levels were not significantly different. Coumarin levels are consistent with levels previously reported to be naturally present in tobacco. The observed positive correlation between PAH and coumarin levels is consistent with fire-cured tobacco being the major source of coumarin in these STPs.

Even at the highest concentrations of coumarin found in the present study, and using published consumption rates, exposure to coumarin from use of STPs is several orders of magnitude below the EFSA recommended safe intake level.

α-AL has been quantified for the first time in tobacco. During method development it was found that α-AL could be readily hydrolysed during extraction and analysis, and precautions were taken to remove water from STP samples. We therefore cannot rule out the possibility that hydrolysis could also occur in the STP during storage. Of the 67 samples analysed most of the STPs did not contain quantifiable levels of α-AL. Three of the STPs had levels of α-AL that were above the LOQ (65 ng/g), and for four others, estimates could be made of their α-AL concentrations which were between the LOD (30 ng/g) and the LOQ. The classification of α-AL as a GRAS flavour component, and its low prevalence and levels in STPs would indicate that it would not present a risk to the consumer.

β-AL was tentatively identified in three of the STPs, but it was not possible to quantify the levels with any accuracy.
